# Perspectives of Vietnamese Americans Regarding COVID-19 Vaccine Acceptance, Trusted Sources of Information, and Pandemic-related Challenges

**DOI:** 10.1007/s40615-025-02327-7

**Published:** 2025-03-08

**Authors:** Celine Nguyen, Ben King, Jannette Diep, Lauren Gilbert, Bich-May Nguyen

**Affiliations:** 1 Vietnamese Culture and Science Association, Houston, TX USA; 2 The University of Texas Southwestern Medical School, Dallas, TX USA; 3 Tilman J. Fertitta Family College of Medicine, The University of Houston, 5055 Medical Circle, Houston, TX USA; 4 Humana Integrated Health Systems Sciences Institute, University of Houston, Houston, TX USA; 5 Boat People SOS Houston, Houston, TX USA; 6 University of Wyoming, Laramie, WY USA

**Keywords:** COVID-19 vaccines, Vaccine hesitancy, Immunization, Vaccination, Vietnamese Americans, Asian Americans

## Abstract

**Background:**

Due to limited research on Asian Americans and COVID-19, we investigated the perspectives of Vietnamese Americans regarding COVID-19 vaccine acceptance, trusted sources of information, and pandemic-related challenges.

**Methods:**

Vietnamese American adult residents in Texas were recruited between September 2021 through March 2022 to complete the NIH CEAL Common Survey 2 electronically in English or Vietnamese, which contains 23 questions about social determinants of health, information, trust, risk perception, testing and disease control, COVID-19 vaccination, and demographics. We analyzed data using bivariate logistic or linear regression models.

**Results:**

Of the 224 completed responses, 181 participants were vaccinated (80.8%), 20 (8.9%) were partially (one of two-dose courses) vaccinated, and nine (4%) were unvaccinated. Of the unvaccinated individuals, 44.4% reported that getting vaccinated in the next 3 months was “not likely at all,” and the top barriers included safety concerns (77.8%), side effects (66.7%), and vaccine efficacy (44.4%). Vietnamese-language responders had significantly higher odds of experiencing non-medical challenges in obtaining food (OR = 2.08, *p* = 0.020) and transportation (OR = 2.56, *p* = 0.008) than English-language responders. Older age was significantly associated with reporting non-medical challenges in obtaining food (*β* = 8.39, *p* < 0.001), water (*β* = 9.58, *p* < 0.05), medications (*β* = 6.43, *p* < 0.05), and transportation (*β* = 5.69, *p* < 0.05).

**Conclusion:**

Our findings describe barriers to vaccine acceptance and reveal variance in the prevalence of non-medical challenges among Vietnamese-language participants. It also showed within-group variation in COVID-19 vaccine attitudes and trusted sources of information among Vietnamese Americans. Research with disaggregated data can guide strategies to address non-medical health disparities in diverse communities.

## Introduction

The COVID-19 pandemic has disproportionately affected people of color in the United States, highlighting the need for comprehensive research investigating how COVID-19 has impacted diverse ethnic groups. Existing studies show that disaggregated COVID-19 data within individual Asian American and Pacific Islander (AAPI) subgroups are not commonly reported, limiting understanding of potential disparities across this heterogeneous population [1]. Preliminary research examining disaggregated data on COVID-19 reveals disproportionately high mortality rates among AAPIs, with Vietnamese individuals exhibiting nearly double the odds of hospitalization related to COVID-19 than non-Hispanic whites and several Asian subgroups [2, 3]. Unfortunately, there is limited research evaluating disaggregated data on the facilitators and barriers of COVID-19 vaccine uptake among Vietnamese Americans.

While some studies have disaggregated data on COVID-19 vaccine willingness in AAPI communities, exploration of community-specific characteristics or reasons associated with vaccine acceptance is limited [4]. Despite a subpopulation exceeding 2.3 million in the United States in 2023, Vietnamese Americans have received modest research attention [5]. In addition to being overrepresented in COVID-19 cases, additional research suggests that Vietnamese Americans who work in high-contact industries face an increased risk of infection [5]. Analyzing the facilitators and obstacles associated with COVID-19 vaccination uptake among Vietnamese Americans could offer valuable insights into actionable strategies for community leaders, government entities, and institutions seeking to engage this specific population effectively.

Identifying individual beliefs and barriers is the crucial first step in promoting health, especially in COVID-19 prevention. Health behaviors, including vaccination acceptance, within the Vietnamese American community are often deeply rooted in Vietnamese culture, and thus can deviate significantly from those of the general population and other minority groups [6]. Cultural differences, personal beliefs, limited English proficiency, and diverse trusted information sources may further influence COVID-19 vaccine access and acceptance, influencing health behaviors and outcomes [7–9]. Exploring these factors can provide insights into the influence of individual beliefs and barriers on COVID-19 vaccine acceptance among the distinct demographic segments of the Vietnamese American community.

Resource-related barriers, such as lack of access to a primary care provider, and education level, can greatly impact health and health prevention behaviors [10]. After the enactment of the Affordable Care Act, the number of uninsured Vietnamese Americans is currently comparable to white Americans [10, 11]. Among the literature on hepatitis B virus (HBV) screening and vaccination behaviors, lack of a regular source of care were associated with fewer HBV screening behaviors among Vietnamese Americans [12, 13]. One study investigating the prevalence and barriers of HBV screening and vaccination among Asian Americans found that while Vietnamese Americans had the lowest rates of screening and vaccination as a subgroup; being younger, having a higher knowledge of HBV, and having health insurance were significantly associated with HBV vaccine uptake rates within the Vietnamese American subgroup [14]. However, the association between similar sociodemographic and person-level factors, including a regular source of care, continued health insurance coverage during the pandemic, and education level, has not yet been explored in patterns of COVID-19 vaccine uptake in this demographic group.

For immigrants, including Vietnamese Americans, social support and social networks are important factors associated with health information-seeking behavior [15, 16]. Studies focusing on Vietnamese Americans have identified that improvements in family and community safety, rooted in collectivist values and aimed at reducing health risks for both family and the broader community, are key facilitators of COVID-19 vaccine uptake [17]. However, further investigations are warranted to expand our understanding of the impacts of collectivism on fostering health promotion behaviors among Vietnamese Americans. Exploring the impacts of a collectivist mindset on vaccine acceptance within this population can offer valuable insights for future interventions and public health initiatives tailored to the nuanced needs of these communities.

To address research gaps among communities of color during the coronavirus pandemic, the National Institutes of Health (NIH) launched the Community Engagement Alliance (CEAL) against COVID-19 disparities to conduct community-engaged research and outreach [18]. Through collaboration with the Texas CEAL Consortium and community-based organizations (CBO), we build upon qualitative work by investigating the attitudes and behaviors of Vietnamese Americans in Texas [8]. Our objective was to investigate facilitators and barriers to COVID-19 vaccine acceptance in Vietnamese Americans in Texas and identify challenges related to non-medical drivers of health. Additionally, we explore the association with trusted sources of information and individual beliefs such as COVID-19 vaccine confidence, collectivism, health insurance status, access to a primary care provider, and education level on COVID-19 vaccine uptake.

## Methods

The NIH CEAL program developed the Common Survey 2 instrument. Tier 1 contained 23 questions covering topics including healthcare access, social determinants of health, information, trust, risk perception, testing and disease control, COVID-19 vaccination, research participation, and demographics (Appendix 1). Participants self-assessed their race and ethnicity to ensure that research participants belonged to the population of interest. On average, the survey took 15 min to complete.

Survey translation was performed by CBO partners with extensive experience in Vietnamese community organizing, including the progressive Vietnamese American organization PIVOT and Boat People SOS-Houston (BPSOSH). First, a PIVOT translation team member translated the survey from English to Vietnamese. Subsequently, a different PIVOT translator back translated the survey from Vietnamese to English. Native Vietnamese speakers at BPSOSH reconciled discrepancies to ensure the survey’s accessibility and understanding to the population of interest.

Inclusion criteria for participants were (1) at least 18 years of age, (2) of Vietnamese ethnicity, (3) living in Texas, and (4) able to read and write in either English or Vietnamese. Participants completed all measures in a Qualtrics survey accessible by web browsers on computers and mobile devices in English and Vietnamese. A successful captcha verification was required before answering survey questions to prevent survey bots. Participants were recruited through a convenience sampling approach between September 20, 2021 and March 4, 2022. Bilingual recruitment efforts included virtual outreach over email listservs, social media posts, and a digital recruitment ad that ran for 3 weeks in January 2022. Additionally, participants were recruited in person during two community health fairs and literacy classes in collaboration with partner organizations. Informational flyers in both English and Vietnamese were posted inside two Houston-area health clinics serving predominately Vietnamese populations.

All participants self-reported meeting the inclusion criteria and provided informed consent online before completing the survey. After submission, participants received a standard debriefing form, including contact information of the research team for any follow-up questions. Participants who provided a valid email address or phone number were entered into a raffle for one of five $50 Visa gift cards. This research was approved by the University of Houston Institutional Review Board (June 4, 2021/STUDY00003046) and performed in accordance with relevant ethical guidelines and regulations.

### Demographic Information

Participants responded to demographic questions characterizing their racial/ethnic identity, age, gender identity, sexual orientation, employment status, language spoken at home, and need for assistance in reading written information from the doctor or drug store. Age was analyzed continuously in this study. Participants responded to questions characterizing how long it has been since they last saw a doctor or healthcare professional about their health, insurance status, healthcare plan, and if health coverage was lost during the COVID-19 pandemic.

### COVID-19 Information and Vaccine Confidence

Participants reported how often they had been tested for COVID-19 and if they had ever tested positive for COVID-19. Participants also reported how many doses of the COVID-19 vaccine they received. If they were not vaccinated for COVID-19, they reported the likelihood of getting a COVID-19 vaccine in the next 3 months on a scale of 1, not likely at all, to 7, extremely likely. If not vaccinated for COVID-19, participants selected reasons for not getting a COVID-19 vaccine. Participants were given the opportunity to write additional comments or concerns regarding COVID-19 vaccination. Additionally, unvaccinated and vaccinated participants reported difficulties, if any, in getting a COVID-19 vaccine. Participants were also given the opportunity to write comments or concerns regarding COVID-19 vaccination barriers. Vaccinated participants reported reasons why they received a COVID-19 vaccine. All participants rated confidence that the COVID-19 vaccines currently available in the United States are safe on a Likert scale of “Not at all confident,” coded as 1, and “Very confident,” coded as 7.

### COVID-19 Vaccine Facilitators

COVID-19 vaccine facilitators refer to factors that positively influenced or supported vaccination uptake. Participants were asked to select all applicable reasons for getting or intending to get a COVID-19 vaccine from a provided list of options (Appendix 1). The options included personal, familial, and community motivations, as well as guidance from healthcare providers and beliefs about vaccine efficacy. Specifically, the facilitators assessed were as follows: keeping oneself, one’s family, or one’s community safe; having a chronic health problem such as asthma or diabetes; being advised by a doctor; avoiding serious illness from COVID-19; feeling safe around others; the belief that normalcy would only return with widespread vaccination; and expectations from one’s community or family. Responses were coded as binary variables (endorsed: yes; not endorsed: no) for analysis. Additionally, participants could select “I would not get a COVID-19 vaccine” or specify other reasons under an open-ended option.

### COVID-19 Vaccine Barriers

COVID-19 vaccine barriers refer to factors negatively associated with vaccination. Participants were asked to select all applicable reasons for not receiving a COVID-19 immunization from a provided list of options (Appendix 1). The options included not liking needles, believing they were at low risk, concerns about effectiveness or safety, community approval, having been infected with COVID-19, religious beliefs, concern about having the right identification card, or contracting COVID-19 from a vaccination site. Another question asked participants about what has made getting a COVID-19 vaccine difficult. Options include worries about payment, location, transportation, time off work, making an appointment, child care, language access, and recognized identification card. Additionally, people who received at least one dose of the COVID-19 vaccine were asked about difficulties in obtaining the immunization. Options included scheduling an appointment, length of the appointment, showing identification, transportation, location, child care, time off work, language access, vaccine safety, worries of contracting COVID-19 at a vaccine site, immunization side effects, effectiveness, needles, religious beliefs, and community approval.

### Non-Medical Challenges

COVID-19 vaccine barriers refer to factors that negatively influenced or supported vaccination uptake. Participants were asked whether they had experienced various challenges related to the COVID-19 pandemic in the past month, regardless of whether they had contracted COVID-19. The survey assessed challenges such as obtaining health care (including mental health), having a place to live, accessing enough food, clean water, or medications, and arranging transportation. Participants indicated the severity of each challenge as “no, this is not a challenge,” “yes, this is a minor challenge,” or “yes, this is a major challenge.” For analysis, responses were dichotomized into two categories: “no challenge” versus “any challenge” (combining minor and major challenges).

### Trust in Information Sources

Participants were asked to rate their level of trust in various sources of information about COVID-19. The sources included their doctor or healthcare provider, faith leader, coworkers or classmates, news media, social media contacts, the federal government, state/local government, tribal leadership, the Centers for Disease Control and Prevention (CDC), and community organizations. Responses were categorized into “Not at all,” “A little,” “A great deal,” “Don’t know,” “Does not apply,” and “Prefer not to answer.” Participants were considered to trust an information source if they reported “A little” or “A great deal” of trust in that source. Participants were also asked to rate their level of trust in the United States Food and Drug Administration to ensure the COVID-19 vaccine is safe for the public and trust in the federal government to ensure a COVID-19 vaccine is safe for children. Responses were categorized into “Do not trust,” “Somewhat trust,” “Mostly trust,” “Fully trust,” and “Prefer not to answer.” Participants were considered to trust if they reported “Mostly trust” and “Fully trust” in that source.

Data analysis was performed in STATA (v17, College Station, TX, USA). Only responses from the population of interest with a 100% completion rate were included in the analysis; however, a “Prefer not to answer” option was commonly available and those responses were treated as missing for analysis purposes. Logistic regressions provided odds ratios to characterize the unadjusted relationships among the primary categorical variables of predisposing demographic, socioeconomic, trusted sources of COVID-19 information, and vaccination measures. The association of age with experiential, attitudinal, and behavioral measures was conducted using linear regression tests, permitting report of the mean differences in years of age associated with differences in categorical independent variables rather than the change in odds for each 1-year increase in respondent age.

## Results

Of the 303 surveys started, 297 surveys were fully completed. Of these completed surveys, 224 respondents identified as Vietnamese Americans in the demographics questions and were included in the data analysis.

### Sociodemographic Characteristics

Table 1 summarizes the sociodemographic characteristics of participants. The mean age was 48.6 years, and the mean household size was four individuals. Of the 167 English-language responders, 83.8% reported speaking Vietnamese at home. Vietnamese-language responders were more likely to require assistance with medical or pharmaceutical instructions (OR = 5.87, 95% CI 2.91–11.86) and were more likely to be elderly (OR = 3.65, 95% CI 1.78, 7.50).

**Table 1:** Sociodemographic characteristics (*N* = 224)

Characteristic		
	*n*	*%*
Gender		
Female	125	55.8
Male	94	42.0
Transgender male or trans male	1	0.5
Nonbinary, genderqueer, or genderfluid	1	0.5
Prefer not to answer	3	1.3
Age		
18 to 29 years	41	18.3
30 to 39 years	37	16.5
40 to 49 years	40	17.9
50 to 64 years	62	27.7
65 years or older	44	19.6
Preferred language to use during the survey		
English	167	74.6
Vietnamese	57	25.4
Insurance status^a^		
Insured	194	87.8
Uninsured	23	10.4
Prefer not to answer	4	1.8
COVID-19 vaccination status		
Fully vaccinated	181	80.8
First dose of the two-dose vaccine	20	8.9
Not vaccinated	9	4.0
Prefer not to answer	14	6.3
Highest level of education		
Less than high school	25	11.2
High school or G.E.D. equivalent	47	21.0
Some college	37	16.5
Associate or college degree	87	38.8
Professional or doctorate degree	21	9.4
Prefer not to answer	7	3.1
Non-medical Challenges (“minor” or “major challenge” combined)		
Getting health care including mental health	125	57.3
Having a place to live	80	37.0
Getting enough food to eat	104	42.3
Having clean water to drink	88	39.8
Getting needed medications	96	44.0
Transportation	143	64.4
Care giving	84	40.8

^a^The sample size for insurance status was due to three missing cases. Participants were given the option not to disclose their insurance status

### COVID-19 Vaccine Confidence

Eighty percent of participants were fully vaccinated, 9% were partially vaccinated, and 6% declined to answer the vaccination status question. Among the nine unvaccinated participants (4%), 44.4% reported that getting vaccinated in the next 3 months was “not likely at all.” Barriers to vaccination among unvaccinated participants included concerns about safety (77.8%), side effects (66.7%), and vaccine efficacy (44.4%).

Participants who rated a 4 or higher out of 7 on the likelihood of getting vaccinated in the next 3 months reported specific barriers, including not knowing how to make an appointment, lack of transportation, and language barriers. Among vaccinated participants, the challenges most commonly reported included delays in securing an appointment (15.3%), distant or inaccessible vaccination locations (9.8%), lack of knowledge about how to make an appointment (9.3%), and concerns about vaccine side effects (8.8%).

Older participants had a positive association with confidence in the safety of COVID-19 vaccines (*β* = 4.80, 95% CI 0.10–9.50). Seventy-five percent of participants trusted the FDA to ensure vaccine safety for the public, 71.9% trusted the FDA to ensure vaccine safety for children, and 57.1% reported feeling very confident in the safety of the vaccines available in the United States.

### COVID-19 Vaccine Facilitators

The most frequently reported reasons for vaccination were a desire to keep oneself safe (72.3%), keep one’s family safe (71.9%), keep one’s community safe (57.1%), and avoid serious illness from COVID-19 (41.1%).

Participants who reported being motivated by family safety had higher odds of being fully vaccinated (OR = 28.55, 95% CI 3.08–263.70) and of expressing confidence in vaccine safety (OR = 2.81, 95% CI 1.47–5.37). Participants motivated by community safety also had higher odds of being fully vaccinated (OR = 5.64, 95% CI 1.11–28.55) and expressing confidence in vaccine safety (OR = 2.80, 95% CI 1.55–5.04). Vietnamese-language responders had higher odds of reporting community safety as a vaccine motivator (OR = 2.11, 95% CI 1.10–4.06) compared to English-language responders, while the odds of reporting family safety as a motivator did not differ significantly by language group. Table 2 provides odds ratios for study variables associated with endorsing family and community safety as vaccine motivators.

**Table 2:** Odds ratios (OR) from bivariate logistic regression tests of rationale for receiving vaccine

Variable	*n*	Endorsed community safety		Endorsed family safety	
		OR	95% CI	OR	95% CI
Vaccinated for COVID-19	210	5.64*	1.11–28.55	28.55*	3.08–263.70
Confidence in safety of COVID-19 vaccine	212	2.79*	1.55–5.04	2.81*	1.47–5.37
Trust in federal government for COVID-19 information	172	2.40*	1.24–4.67	1.74	0.83–3.68
Trust in the centers for disease control and prevention (CDC) for COVID-19 information	198	2.10*	1.09–4.04	1.88	0.94–3.80
Trust in social media for COVID-19 information	183	0.96	0.49–1.91	0.48*	0.23–0.99
Challenge accessing food	220	0.71	0.41–1.22	0.55*	0.30–0.99

The odds ratio for study variables and (1) endorsing community safety as a facilitator to get the COVID-19 vaccine and (2) endorsing family safety as a facilitator to get the COVID-19 vaccine

*Statistically significant

### Non-Medical Challenges

Many participants reported experiencing challenges related to COVID-19, with the most frequently cited issues being transportation (63.8%), health care (55.8%), food (46.4%), medications (42.9%), clean water (39.3%), housing (35.7%), and care for dependents (37.5%). Vietnamese-language responders had higher odds of reporting challenges obtaining food (OR = 2.08, 95% CI 1.11–3.89), water (OR = 2.73, 95% CI 1.45–5.14), and transportation (OR = 2.56, 95% CI 1.25–5.28) compared to English-language responders.

Older age was positively associated with reporting challenges obtaining food (*β* = 8.39, 95% CI 3.98–12.80), water (*β* = 9.58, 95% CI 5.12–14.04:), medications (*β* = 6.43, 95% CI 1.88–10.98), and transportation (*β* = 5.69, 95% CI 1.02–10.36). College-educated participants had lower odds of reporting challenges obtaining transportation (OR = 0.50, 95% CI 0.28–0.88).

### Trusted Sources of Information

**Fig. 1:**
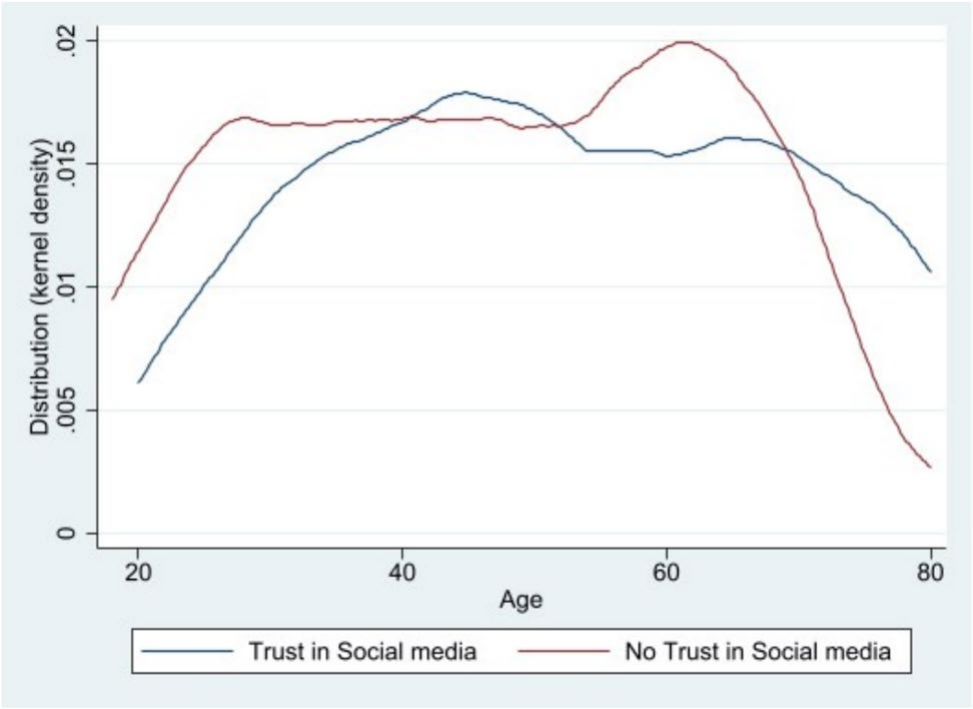
Age distribution stratified by trust in social media for COVID-19 information. This distribution graph highlights the relationship between age in years and trust in social media for COVID-19 information. Individuals that did not trust in social media for COVID-19 information (*n* = 179) had a median age of 46 years, with an IQR of 32–62 years. Individuals that trusted social media for COVID-19 information “a great deal” (*n* = 45) had a median age of 52 years, with an IQR of 37–70 years. This data is from an online survey for Vietnamese adults living in Texas that collected information from September 2021 to March 2022

Most participants trusted the FDA to ensure vaccine safety for the public (75.9%) and for children (71.9%). However, 57.1% reported feeling very confident in the safety of vaccines currently available in the United States. Social media trust was associated with older age (*β* = 7.42, 95% CI 1.61–13.23) (Fig. 1). The median age of participants who trusted social media was 52 (IQR = 37–70), compared to 43 (IQR = 30–61) among those who did not. College-educated participants had higher odds of trusting the federal government (OR = 2.16, 95% CI 1.14–4.09), while non-college-educated participants had higher odds of trusting social media (OR = 0.47, 95% CI 0.23–0.94). Table 3 provides detailed trust levels for various sources of COVID-19 information.

**Table 3:** Trust in sources for COVID-19 information

Source		Likert scale *N*, %				
	*n*	Not at all	A little	A great deal	Don’t know	Does not apply
Your doctor or healthcare provider	221	17 (7.6%)	27 (12.1%)	161 (71.9%)	13 (5.8%)	3 (1.3%)
Your faith leader (e.g., priest, minister, rabbi)	219	56 (25.0%)	48 (21.4%)	49 (21.9%)	38 (17.0%)	28 (12.5%)
People you go to work or class with or other people you know	221	38 (17.0%)	89 (39.7%)	49 (21.9%)	34 (15.2%)	11 (4.9%)
News on the radio, TV, online, or in newspapers	220	25 (11.2%)	76 (33.9%)	98 (43.8%)	16 (7.1%)	5 (2.2%)
Your contacts on social media	221	52 (23.2%)	86 (38.4%)	45 (20.1%)	30 (13.4%)	8 (3.6%)
The federal government	217	32 (14.3%)	70 (31.3%)	70 (31.3%)	39 (17.4%)	6 (2.7%)
State and/or local government	218	34 (15.2%)	75 (33.5%)	68 (30.4%)	36 (16.1%)	5 (2.2%)
Tribal leadership	215	63 (28.1%)	24 (10.7%)	13 (5.8%)	65 (29.0%)	50 (22.3%)
CDC	219	14 (6.3%)	37 (16.5%)	147 (65.6%)	17 (7.6%)	4 (1.8%)
A community organization that provides services and assistance where you live	217	27 (12.1%)	76 (33.9%)	70 (31.3%)	30 (13.4%)	14 (6.3%)

This table highlights the trust in sources for COVID-19 information using a 3-point Likert scale. “Don’t know” and “Does not apply” were included as response options

## Discussion

Identifying barriers and facilitators of vaccine uptake in the Vietnamese American community is essential in developing health communication strategies. Evidence-informed approaches are needed for drafting vaccination outreach campaign messages as COVID-19 becomes endemic, new surges in cases arise, and updated vaccines become available. Our findings of concerns about the COVID-19 vaccine’s safety, side effects, and efficacy as barriers to vaccination support previous findings from studies on Asian Americans [4] and the general US population [2]. Another study on Vietnamese Americans also found that long wait times at vaccination centers, distance to vaccine distribution center locations, and fears about misinformation about the efficacy and safety were initial barriers to getting the COVID-19 vaccine [8]. Research conducted in Chicago that identified that COVID-19 vaccination is hampered by structural barriers like distance in addition to vaccine hesitancy supported these findings [19]. Comparatively, diverse studies investigating global vaccine hesitancy rates and barriers found that the most reported barriers to childhood COVID-19 vaccination included fear of side effects, mothers’ lower education level, financial instability, and low confidence in new vaccines [20, 21]. Addressing barriers, emphasizing the collective good, and promoting perceived personal and societal benefits could improve vaccine confidence among the Vietnamese American population.

The sample’s most reported trusted sources for COVID-19 information included the participants’ doctor or healthcare provider, the CDC, news outlets, the federal government, and community organizations. This finding is consistent with a study focusing on Vietnamese Americans and another diverse study with a majority of Black respondents that found the most trusted sources of information included medical professionals, community organizations, and the government [17, 22]. Comparatively, a study of US adults (not disaggregated by race or ethnicity) found that the most trusted sources of COVID-19 information were from state health departments, the CDC, and a university [23].

We found that risk factors for reporting challenges related to COVID-19 included older age, being uninsured, not using English as the preferred language, and not being college-educated. Our findings that older Vietnamese adults were more likely to experience non-medical challenges align with prior research showing that older populations have been disproportionately affected by the pandemic [24]. Of the non-medical challenges, obtaining food and water was the most reported among older adults in our survey. Previous research on food insecurity has demonstrated age as a risk factor [25]. Studies report that the Vietnamese subgroups have one of the highest prevalence of food insecurity among Asian American populations [26]. Having adequate access to food is a basic human need, and malnourishment and undernourishment can be related to chronic health conditions.

Public health and healthcare systems are often centered on English speakers, which can create systemic barriers to health, such as miscommunication between healthcare professionals and patients and increased costs and length of treatment for patients who speak other languages [26]. Our survey found that almost 65% of Vietnamese-language responders reported needing someone to help read written information from the doctor or drug store. Additionally, a recent study found that language barriers experienced by Asian Americans impact both their healthcare decisions and treatment choices [27]. The same study found that language facilitators enhanced the connection between Asian Americans and the healthcare system [27]. Because nearly half of Vietnamese Americans report limited English proficiency, they often face language barriers when accessing health care in the United States [9]. Providing information related to health and available social services in preferred languages is necessary to improve access to care.

Although educational inequalities in COVID-19-specific challenges have not yet been extensively reported, many studies identified the impact of education on health and longevity [28]. Our findings correlate to literature highlighting those individuals with higher educational attainment live healthier lives than their less educated peers [28, 29]. They also correlate to studies focusing on the relationship between education levels and HBV vaccine uptake in Vietnamese Americans [13, 29]. Tailored outreach efforts inclusive of language and at appropriate education levels to increase awareness of and access to resources are essential.

We investigated the perspectives of Vietnamese Americans regarding COVID-19 vaccine acceptance, trusted sources of information, and pandemic-related challenges. This study represents the first comprehensive quantitative survey conducted on this specific population, shedding light on the unique challenges and attitudes they have encountered during these unprecedented times. By focusing on an under-researched community, we have gathered disaggregated data, which is crucial for understanding the impact of COVID-19 on Vietnamese Americans. Through our investigation, we identified factors that impact health-prevention behaviors within this population, particularly in relation to age, language, and educational background. Additionally, we provided detailed insights into variations in attitudes, behaviors, and trusted sources of information among different subgroups within the Vietnamese American community.

This study has several potential limitations. First, this cross-sectional survey design describes correlations but not causation. Second, the research team experienced challenges in fielding this survey. To prevent bots, participants were required to complete a captcha verification before answering survey questions. However, our community partners reported that older adults had difficulties completing this verification. Future research should explore other means to promote sample representation while addressing these accessibility concerns. Third, our community partner reported challenges in promoting the survey to unvaccinated individuals, many of whom were hesitant to participate. Since there were a small number of unvaccinated participants, we chose to report the findings, but did not analyze associations. Investigations into promoting trust in public health research among this group are necessary to capture their attitudes and behaviors to promote vaccine uptake. Additionally, the survey was conducted online, drawing participants with a stable internet connection, of which three quarters of respondents used English. Thus, these results may not be generalizable. Nonetheless, this study is one of the few that explores COVID-19 vaccine acceptance and the impact of the disease among Vietnamese Americans during the pandemic.

These findings provide preliminary insights into the perceptions of the COVID-19 vaccine and trusted sources of information among the Vietnamese American adult population in Texas. We identified barriers to COVID-19 vaccine uptake, including concerns about the COVID-19 vaccine’s safety, side effects, and efficacy, and found that COVID-19 vaccine facilitators included a desire to keep the family, self, and community safe. The most trusted sources for COVID-19 information were the participants’ doctor or healthcare provider, the CDC, news outlets, the federal government, and community organizations. Our study revealed the risk factors of age, language, and education for non-medical challenges among Vietnamese Americans. Underscoring our findings is the need for culturally and linguistically competent strategies for providing health outreach and services to the Vietnamese American population.

Future studies could examine vaccine concerns in a larger and more diverse sample of Vietnamese Americans, including unvaccinated people, to examine if these results are replicable. In addition, reports from community partners on the slow uptake of COVID-19 boosters and COVID-19 vaccinations among children highlight the need to investigate the behaviors, attitudes, and barriers of additional doses and immunizations among parents and children. Differences in trusted sources related to age, education level, and endorsement of collectivism reveal the need to explore effective and diverse outreach strategies to improve health outcomes among Vietnamese Americans and their distinct demographic subgroups.

## Data Availability

The raw data are protected and unavailable due to data privacy laws. The processed data sets are available upon request.
